# Genome-Wide Identification of the *OPR* Gene Family in Soybean and Its Expression Pattern Under Salt Stress

**DOI:** 10.3390/biology15010032

**Published:** 2025-12-25

**Authors:** Zhongxu Han, Xiangchi Zhang, Yanyan Sun, Chunjing Lin, Xiaoyang Ding, Hao Yan, Yong Zhan, Chunbao Zhang

**Affiliations:** 1 Faculty of Agronomy, Jilin Agricultural University, Changchun 130118, China; han13766616247@163.com; 2 Soybean Research Institute, Jilin Academy of Agricultural Sciences (Northeast Agricultural Research Center of China), Changchun 130033, China; sunyy@jaas.com.cn (Y.S.); lincj@jaas.com.cn (C.L.); dingxy_s@163.com (X.D.); yanhaoonline@163.com (H.Y.); 3 Crop Research Institute, Xinjiang Academy of Agricultural and Reclamation Sciences, Shihezi 832000, China; 17699537028@163.com

**Keywords:** soybean, 12-oxo-phytodienoic acid reductase, salt stress

## Abstract

12-oxo-phytodienoic acid reductase (OPR) is a core component of the jasmonic acid (JA) biosynthetic pathway and participates in JA synthesis by catalyzing the reduction in the precursor 12-oxo-phytodienoic acid (OPDA), as well as broadly regulating plant development, stress response, and hormone signaling networks. This study analyzed the *OPR* gene family using 28 soybean genomes. A total of 15 *OPR* gene family members in soybean were identified, including 14 core genes and one variable gene. Analysis of gene duplication types showed that whole-genome duplication (WGD)/segmental duplication was the main mode of duplication in *GmOPR*s. The phylogenetic tree constructed from multiple species showed that the *OPR*s in subgroup VII were functionally important *OPR* genes and that the *OPR*s underwent *Leguminosae* and *Cruciferae* divergence, and large-scale duplication occurred in *Leguminosae*. Analysis of natural selection pressures on 28 soybean accessions indicated that the overall evolutionary pressures on *GmOPR*s were dominated by purifying selection, but there were also potential positive selection signals. Analysis of cis-acting elements revealed a large number of light- and hormone-responsive cis-acting elements in the *GmOPR*s. Some specific cis-acting elements were only present in a few genes or accessions. The protein interaction network consisted of 12 GmOPR proteins, 4 allene oxide synthase (AOS) proteins, and 6 allene oxide cyclase (AOC) proteins, where AOCs interact with GmOPRs and AOSs. Tissue transcriptome expression profiling showed that *GmOPR3*, *GmOPR7*, and *GmOPR15* were specifically expressed in roots, whereas *GmOPR2*, *GmOPR10*, and *GmOPR14* were specifically expressed in leaves, suggesting that these genes play an important role in the growth and development of the tissues. Moreover, *GmOPR*s usually responded to salt stress, and *GmOPR3*, *GmOPR8*, *GmOPR9*, *GmOPR10*, *and GmOPR11* were significantly up-regulated in roots and leaves under salt stress. This suggests that these genes may be involved in biological processes such as osmoregulation, ion homeostasis, and scavenging of reactive oxygen species, thus helping soybeans to resist salt stress. This study comprehensively analyzed the *OPR* gene family in soybean based on the 28 soybean accessions and clarified the salt stress response pattern, which provides a new and more effective and reliable way to analyze the soybean gene family.

## Simple Summary

Soybean (*Glycine max* (L.) Merr.) is an important oilseed crop in the world, but its yield is severely affected by salt stress. Jasmonic acid is a hormone closely related to plant growth, development, and response to adversity, and the 12-oxo-phytodienoic acid reductase (OPR) is a key enzyme in the jasmonic acid synthesis pathway. In order to characterize the *OPR* gene family in soybean and screen for key response genes under salt stress, members of the *OPR* gene family were identified and analyzed in three wild soybean accessions, nine local accessions, and sixteen cultivated accessions. We also investigated the response pattern of the *GmOPR* gene family under salt stress, and the results showed that the *GmOPR* gene family widely responded to salt stress, and *GmOPR3*, *GmOPR8*, *GmOPR9*, *GmOPR10*, and *GmOPR11* were strongly up-regulated in both roots and leaves under salt stress. This study provides a theoretical basis for further understanding of the structure and function of the *GmOPR* gene family and provides candidate genes with application value for soybean stress tolerance breeding.

## 1. Introduction

Jasmonic acid (JA) and its derivatives are an important class of lipid hormones ubiquitously present in plants and are directly involved in the regulation of plant growth and development, such as root elongation [1,2,3], anther dehiscence [4,5], fruit ripening [6,7], and leaf senescence [8,9]. In addition, JA can be used as a signaling molecule for long-distance transport in plants. It participates in responses to biotic stresses, such as pathogen defense [10] and insect pest resistance [11], as well as abiotic stresses, such as cold and freezing tolerance [12,13], heat tolerance [14], drought tolerance [15], and salt tolerance [16]. During these processes, JA usually acts synergistically or antagonistically with other hormones [13,14,17,18].

The biosynthesis of jasmonic acid begins with the degradation of membrane lipids in chloroplasts and the release of α-linolenic acid (α-LeA, 18:3) and hexadecatrienoic acid (HTA, 16:3) by phospholipase [19]. In the successive action of lipoxygenase (LOX), allene oxide synthase (AOS), and allene oxide cyclase (AOC), 12-oxo-phytodienoic acid (OPDA) and its 16-carbon analog, 2,3-dinor-12-oxo-10,15(Z)-phytodienoic acid (dnOPDA), are, respectively, generated [20]. OPDA and dnOPDA are subsequently translocated to the peroxisome, where OPDA is reduced by the OPDA reductase 3 (OPR 3) to 8-(3-oxo-2-(pent-2-enyl)cyclopentyl)octanoic acid (OPC-8) and undergoes three rounds of β-oxidation to produce 6-(3-oxo2-(pent-2-enyl)cyclopentyl)hexanoic acid (OPC-6), 4-(3-oxo-2(pent-2-enyl)cyclopentyl)butanoic acid (OPC-4), and finally JA [21]. This α-LeA -OPDA-JA pathway catalyzed by OPR 3 is considered the primary JA synthesis pathway. However, there also exists an OPR 3-independent pathway for JA biosynthesis in plants, where OPDA can produce dnOPDA through β-oxidation. dnOPDA can generate tnOPDA and 4,5-ddh-JA through two rounds of β-oxidation, which is then reduced to JA by OPR 2 [22]. In addition, dnOPDA and tnOPDA can also generate OPC-6 and OPC-4 by OPR 3, followed by β-oxidation to JA [19]. And JA can be catalyzed by jasmonoyl amino acid conjugate synthase (JAR1), which directly binds to isoleucine to generate jasmonic acid-isoleucine conjugate (JA-Ile), which is translocated to the nucleus, where it binds to the F-box protein COI1 to form a ubiquitin ligase complex, which degrades the jasmonate ZIM-domain (JAZ) repressor proteins, thereby activating the expression of JA-responsive genes such as defense-related genes [23]. Similarly, JA can be chemically modified and metabolized into different compounds such as methyl-JA (MeJA), jasmonyl-ACC (JA-ACC), and 12-O-glucopyranosyl-jasmonic acid (12-O-Glc-JA) [24,25].

OPR belongs to the old yellow enzyme (OYE) family, which is a flavin mononucleotide (FMN)-dependent oxidoreductase [26]. As a core enzyme for jasmonic acid (JA) biosynthesis, OPR participates in JA synthesis by catalyzing the reduction of the precursor OPDA and also broadly regulates plant development, stress response, and hormone signaling networks. Its loss of function leads to severe developmental defects in both haploid and polyploid crops, with the *OPR7/OPR8* double mutant in maize exhibiting male sterility, abnormal flowering, delayed leaf senescence, and increased susceptibility to insect pest pathogens [27], and *OsOPR7* knockdown in rice similarly triggering sterility and flowering disorders [28,29]. In the regulation of salt tolerance, *OPR* members function independently of the JA pathway through abscisic acid (ABA)-dependent pathways, and *TaOPR1* in wheat [30], *ZmOPR1* in maize [31], and *AhOPR6* in peanut [32] were induced by salt stress and overexpressed in *Arabidopsis thaliana* to enhance their salt tolerance, and the mechanism of which may rely on the activation of the ABA signaling pathway, and thus enhance the ability of reactive oxygen species (ROS) scavenging [30]. In addition, the monocot-specific OPRIII subfamily (e.g., wheat) regulates root plasticity through a dose effect. Loss-of-function mutations lengthen the primary root to enhance drought adaptation, whereas overexpression promotes early lateral root emergence and accumulation of JA, which directly correlates with yield under water stress [33]. In summary, *OPR* genes cross-interact with ABA signaling through the main pathway of JA synthesis to synergistically regulate development, stress tolerance, and defense networks, and their functions show significant differentiation among species. Although *OPR* genes have been intensively studied in a variety of plants, there is a paucity of research in soybeans.

Intergenomic comparisons have demonstrated extensive genetic diversity between wild and cultivated soybeans and among cultivated soybeans from different geographic regions [34,35]. Gene family analysis of multiple varieties within a species offers significant advantages in the study of genetics and serves as a powerful method to comprehensively resolve the full genetic diversity of a species. It breaks through the limitations of a single reference genome by integrating genomic data from multiple individuals or varieties to fully reflect the genetic diversity of a population. The legume genus *Glycine* is divided into two subgenera, *Soja* and *Glycine,* and the subgenus *Soja* contains two annual species of wild soybean (*Glycine soja* Siebold & Zucc.) and cultivated soybean (*Glycine max* (L.) Merr.) [36]. With the construction of genome resource libraries of subgenus *Soja* covering different types of wild, farmed, and cultivated species based on structural variation maps from 2898 material genomes and RNA-seq data from 26 representative materials [37], it provides a good platform for in-depth study of functional genomics in soybean and enables genome-wide analysis of gene families from multiple species in the *soja* subgroup.

In this study, members of the *OPR* gene family were characterized in three wild soybean accessions, nine local accessions, and sixteen cultivated accessions. The members were systematically identified and optimized to ensure the accuracy of the gene family analysis. The analysis encompassed 14 core genes and one variable gene, including a novel gene previously undetected using single-reference genome approaches. We investigated the presence/absence variation (PAV) of *GmOPR*s in different soybean accessions, *GmOPR*s localization and distribution patterns, syntenic relationships, and gene duplication events; constructed a phylogenetic tree of *OPR* genes in *Glycine max*, *Oryza sativa*, *Arabidopsis thaliana*, and *Medicago truncatula*; and analyzed the evolutionary relationships; and also analyzed the gene structures, conserved motifs, and the natural selection pressures among soybean accessions. We further predicted and analyzed the cis-acting elements of *GmOPR*s in 28 soybean accessions, the protein interaction network of *GmOPR*s, and the transcriptome expression profiles of *GmOPR* family members. The expression patterns of 15 *GmOPR*s in roots and leaves of soybean under salt stress were detected by quantitative real-time PCR (qRT-PCR), and the *GmOPR*s related to salt stress were screened out in order to understand the potential functions of *GmOPR*s in the JA-dependent salt-responsive pathway and to provide a new idea for the analysis of the soybean gene family in a more effective and reliable way.

## 2. Materials and Methods

### 2.1. Identification of OPR Genes in the 28 Soybean Accessions

The genomic sequences of 26 soybean accessions and the ZH13.v2 accession were downloaded from the SoyOmics database (https://ngdc.cncb.ac.cn/soyomics/, accessed on 21 December 2025) [37], while *Oryza sativa* (*Oryza sativa* v7.0), *Glycine max* (*Glycine max* Wm82.a4.v1), *Arabidopsis thaliana* (*Arabidopsis thaliana* Araport11), and *Medicago truncatula* (*Medicago truncatula* Mt4.0v1) genomes were obtained from the Phytozome Plants database (https://phytozome-next.jgi.doe.gov/, accessed on 21 December 2025) [38]. The *OPR* family HMM profile (PF00724) was retrieved from the PFAM database (http://pfam-legacy.xfam.org/, accessed on 21 December 2025) [39] and used for screening candidate *OPR* proteins with the HMMER software (version 3.2.1), with a threshold of e < 1 × 10^−5^. The re-annotation of gene deletions and protein sequences that contain evident errors is achieved by the interception of the upper and lower 10 kb of chromosomal homologous regions using Softberry (http://www.softberry.com/berry.phtml?topic=fgenesh&group=programs&subgroup=gfind, accessed on 21 December 2025) [40]. The NCBI Batch Web CD-Search Tool (https://www.ncbi.nlm.nih.gov/Structure/bwrpsb/bwrpsb.cgi, accessed on 21 December 2025) [41,42,43] was used to further confirm the reliability of the *OPR* domain prediction in each candidate protein sequence. The presence and absence of *OPR* genes across the 28 accessions were visualized using TBtools software (version 2.119) [44].

### 2.2. Synteny Relationship and Gene Duplication Analysis

The *OPR* gene duplication types and syntenic relationships were analyzed with MCScanX [45], and the gene density and GC ratio for each chromosome were calculated and Circos plotted by TBtools software (version 2.119) [44]. The pie chart of gene duplication events was created by OriginPro 2024 (version 10.1.0.178).

### 2.3. Gene Structures and Motif Patterns

The gene structures were depicted by TBtools software (version 2.119) [44] with the GFF3 file of the ZH13 genome. The conserved motifs scanning of *GmOPR* proteins was conducted by MEME v5.5.7 (https://meme-suite.org/meme/tools/meme, accessed on 21 December 2025) [46], with 10 MEME motifs shown in the result. The visualization of the MEME motifs was created by TBtools software (version 2.119) [44].

### 2.4. Phylogenetic Analysis

The *OPR* family HMM profile (PF00724) was retrieved from the PFAM database (http://pfam-legacy.xfam.org/, accessed on 21 December 2025) [39]. And by HMMER software (version 3.2.1) with a threshold of e < 1 × 10^−5^ in *Oryza sativa* (*Oryza sativa* v7.0), *Arabidopsis thaliana* (*Arabidopsis thaliana* Araport11), and *Medicago truncatula* (*Medicago truncatula* Mt4.0v1) genomes to screen for candidate *OPR* proteins. The NCBI Batch Web CD-Search Tool (https://www.ncbi.nlm.nih.gov/Structure/bwrpsb/bwrpsb.cgi, accessed on 21 December 2025) [41,42,43] was used to further confirm the reliability of the *OPR* domain prediction in each candidate protein sequence of each species. Multiple sequence alignments of *OPR* protein sequences from *Glycine max*, *Arabidopsis thaliana*, *Oryza sativa*, and *Medicago truncatula* were carried out using MUSCLE [47]. Following this, a maximum likelihood (ML) phylogenetic tree was constructed using MEGA 11 software [48] with the JTT substitution model and refined using the iTOL online tool (https://itol.embl.de, accessed on 21 December 2025) [49]. A total of 1000 bootstrap replications were performed to evaluate node support. The *GmOPR*s intraspecific evolutionary tree was constructed using the same method.

### 2.5. Ka/Ks Calculation

The protein and coding sequences (CDS) of *GmOPR* genes in 28 soybean genomes were compared, and Ka/Ks values were calculated using the Ka/Ks Calculator in TBtools software (version 2.119) [44]. The OriginPro 2024 (version 10.1.0.178) was used to create the column line plot of Ka/Ks values. The heatmap of Ka/Ks values for different accessions was plotted using TBtools software (version 2.119) [44].

### 2.6. Analysis of Cis-Acting Elements

The promoter regions of the *OPR* genes were obtained by extracting 2000 bp of genomic sequence upstream of the *OPR* genes in 28 soybean accessions. The cis-acting elements within these promoters were analyzed using PlantCARE (https://bioinformatics.psb.ugent.be/webtools/plantcare/html/, accessed on 21 December 2025) [50]. They were visualized using R software (version 4.3.1) and OriginPro 2024 (version 10.1.0.178).

### 2.7. Protein Interaction Network Analysis

The *OPR* protein sequences of ZH13 were entered into the STRING database (https://cn.string-db.org/, accessed on 21 December 2025) [51] for the construction of a protein interaction network, and the minimum required interaction score was set to high confidence (0.85), with the maximum number of interactors to show set to no more than 5 interactors for both the 1st and 2nd shells.

### 2.8. Analysis of Tissue-Specific Expression Patterns

Tissue transcriptome data for the *OPR* genes of 28 soybean accessions were downloaded from the SoyOmics database (https://ngdc.cncb.ac.cn/soyomics/transcriptome/tissues, accessed on 21 December 2025) [37], including stem, leaf, flower, seed, and root. A representative set of tissue transcriptome data was selected for each *GmOPR* gene and used to plot an expression pattern heatmap with TBtools software (version 2.119) [44].

### 2.9. Expression Patterns of GmOPRs Under Salt Stress

The soybean reference variety Williams 82 was used to analyze expression patterns at different time periods under salt stress. Seeds were placed on filter paper soaked in sterile water and incubated in the dark until seed germination in an incubator at a constant temperature of 26 °C and 65% relative humidity. Then they were transferred to sterilized vermiculite and irrigated with 1/4 Hoagland nutrient solution under the incubation conditions of 26 °C constant temperature, 65% relative humidity, and 14 h of light per day. When the growth reached the V1 developmental period, the control group was kept irrigated with 1/4 Hoagland nutrient solution, while the treatment group was added with 1/4 Hoagland nutrient solution containing 200 mM NaCl for 6, 12, and 24 h. Roots and leaves of plants under 0, 6, 12, and 24 h salt stress treatments were taken, each containing three biological replicates.

All samples were immediately snap-frozen in liquid nitrogen for subsequent RNA extraction using the EasyPure Plant RNA Kit (Cwbio, Beijing, China). Reverse transcription was performed using the TransScript All-in-One First-Strand cDNA Synthesis SuperMix for qPCR (Trans, Beijing, China). qRT-PCR was performed using the 2 × SYBR Green qPCR Mix kit (Trans, Beijing, China) based on the QuantStudio 6 Pro Real-Time PCR System (Thermo Fisher Scientific, Hercules, CA, USA) to detect the expression levels of *GmOPR*s. qRT-PCR was performed using the following reaction programs: 95 °C for 30 s, 40 cycles of 95 °C for 15 s to 60 °C for 1 min, 95 °C for 15 s, 60 °C for 1 min, and 95 °C for 1 s. *Cons4* (BU578186) was used as an internal reference gene, and all the primer sequences used in the experiments are attached in Table S9. The relative expression levels were calculated using the 2^−ΔΔCT^ method [52].

GraphPad Prism 10 (version 10.1.2) was used to analyze and graph qRT-PCR data. Standard deviation and one-way ANOVA were employed to assess significant differences between treatments. Significant differences between control and treatment groups were evaluated using Student’s t-test. The result of *p* < 0.05 was used as the significance threshold.

## 3. Results

### 3.1. Distribution, Synteny, and Duplication Events of GmOPRs in the 28 Soybean Accessions

**Figure 1:**
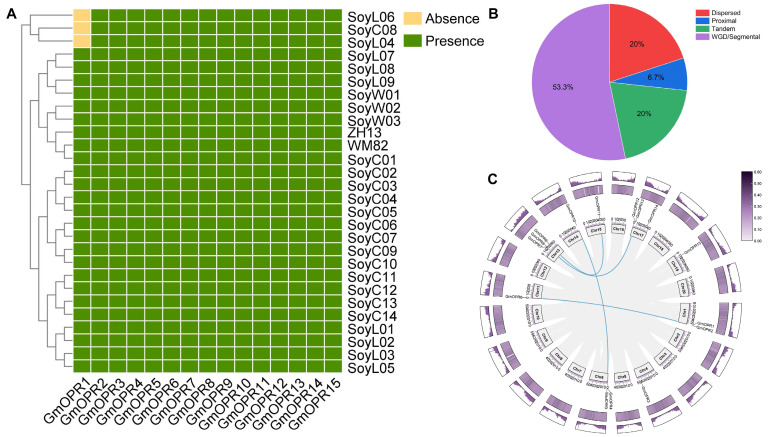
*GmOPR*s in the 28 soybean accessions: presence/absence variants, duplication events, and synteny. (**A**) Presence/absence variants of *GmOPR*s across 28 accessions. (**B**) Distribution of duplication events within *GmOPR*s. (**C**) Synteny analysis and chromosomal distribution of *GmOPR*s. The blue line represents colinear gene pairs within *GmOPR*s. The inner heatmap shows GC ratio, while the outer bar chart indicates gene density.

To identify *OPR* genes in the 28 soybean accessions, candidate genes were identified in 28 soybean genomes using the *OPR* domain (PF00724), and the top and bottom 10 kb of the homology region were intercepted and re-annotated for the genes that were not identified and genes that had obvious errors in the protein structure in different accessions. A total of 105 genes were corrected, including 69 unannotated genes in the 28 soybean accessions, and 36 misannotated genes were re-annotated. Fifteen *OPR* genes were identified in the whole soybean genome; the proteins had lengths between 62 and 459 amino acids (Table S1). Following the criteria proposed in a previous study [53], based on their level of conservation, 14 *OPR* genes were present in 28 accessions and recognized as core genes. The absence of *GmOPR1* in SoyC08, SoyL04, and SoyL06 was identified as a variable gene, and the presence/absence variants (PAV) profile of *GmOPR*s in different soybean accessions indicated that the *OPR* gene family is conserved in soybean (Figure 1A). Notably, *GmOPR6* and *GmOPR9* were translocated in W02, with *GmOPR6* moving to a different position on the same chromosome and *GmOPR9* moving from chromosome 13 to chromosome 11 (Table S1). Except for *GmOPR6* and *GmOPR9* in W02, the same *OPR* genes in different accessions are located in the same region of the chromosome. Most *OPR*s are distributed at both ends of the chromosome in a region with high gene density and moderate GC ratio (Figure 1C). In addition, four pairs of genes belonging to the *GmOPR* gene family were found to be in syntenic relationships. The analysis of gene duplication types can deepen the understanding of the evolutionary pattern of *GmOPR*s. WGD/segmental duplication is the main duplication mode of *GmOPR*s. *GmOPR5*, *GmOPR8*, and *GmOPR13* are tandem duplications; *GmOPR3*, *GmOPR14*, and *GmOPR15* are dispersed duplications; and only *GmOPR2* is a proximal duplication (Figure 1B and Table S2).

### 3.2. Gene Structure, Conserved Motifs, and Phylogenetic Analysis

**Figure 2:**
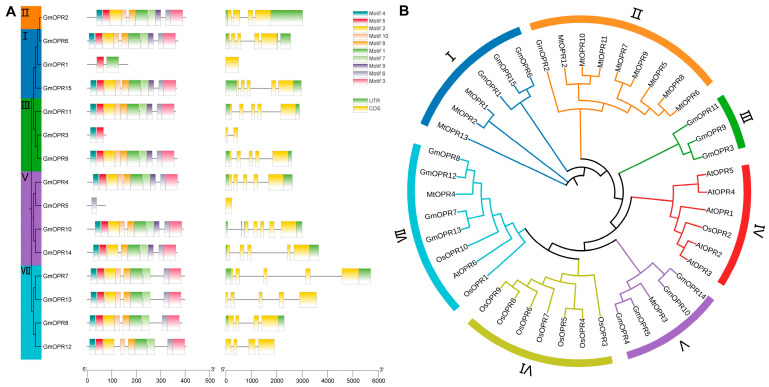
Gene structure, conserved motifs, and phylogenetic tree of the *GmOPR* gene family. (**A**) Gene structure and conserved motifs of the *GmOPR* gene family. The maximum likelihood phylogenetic tree comprising 15 *GmOPR* protein sequences constructed using the JTT model and 1000 bootstraps. Different colors classify different subgroups. (**B**) A phylogenetic tree constructed from the *OPR*s of *Glycine max*, *Oryza sativa*, *Arabidopsis thaliana*, and *Medicago truncatula*. The tree was constructed using the maximum likelihood method with the JTT model and 1000 bootstraps. Different colors classify the branches of the phylogenetic tree.

The analysis of the *GmOPR* gene structure and conserved motifs can provide a deeper understanding of the genetic conservation and functional diversity of the *GmOPR* gene family. Based on a maximum likelihood phylogenetic tree consisting of 15 GmOPR protein sequences constructed with the JTT model and using 1000 bootstraps, the gene structure and motif patterns of the *GmOPR*s were visualized and analyzed using TBtools software. Ten motifs of *GmOPR*s were identified by the MEME online tool, whose length ranged from 21 to 50 amino acids (Table S3). Motif5 was the most predominant motif, contained in 93% of *GmOPR* proteins, whereas Motif9 was the least predominant motif, contained in only 53% of *GmOPR* proteins, and Motif9 was generally missing in subgroup VII with a conserved composition, which may be related to the functional differences in GmOPRs. The number of exons in *GmOPR*s varied from 1 to 5 (Figure 2A).

To reveal the evolutionary relationship and functional evolution of *OPR* genes, a phylogenetic tree was constructed using the maximum likelihood method with the JTT model and using 1000 bootstraps, based on 15 *GmOPR* protein sequences, 13 *MtOPR* protein sequences, 6 *AtOPR* protein sequences, and 10 *OsOPR* protein sequences, and categorized them into subgroups I–VII (Figure 2B and Table S4). Subgroup VII included *OPR* genes from four species, indicating that these *OPR* genes are evolutionarily conserved and play important roles in plant growth and development. Moreover, subgroup VII contains the identified *OPR3* genes of *Arabidopsis thaliana* [54,55] and rice [56], and *GmOPR7*, *GmOPR8*, *GmOPR12*, and *GmOPR13* in the same group may be the *OPR3* genes of soybean. The *OPR* genes of rice and soybean formed independent branches, subgroup III and subgroup VI, which may be due to the fact that both underwent an ancient polyploidization event. Most of the *MtOPR*s and *GmOPR*s were concentrated in the same subgroup and were significantly more numerous than *AtOPR*s, suggesting that the *OPR* genes may have undergone legume and cruciferous differentiation and large-scale duplication in the legume.

### 3.3. Selective Pressure Analysis of GmOPRs in the 28 Soybean Accessions

**Figure 3:**
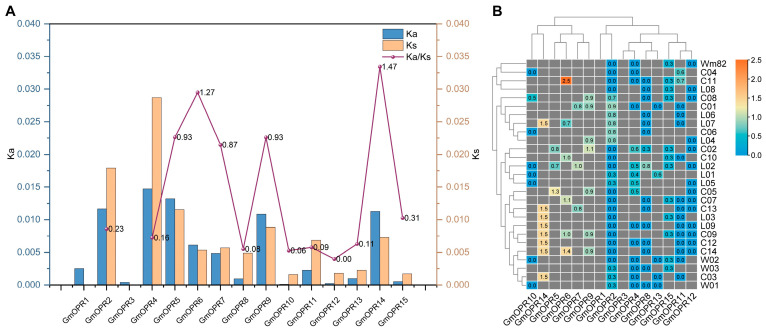
Analysis of natural selection pressures in the 28 soybean accessions. (**A**) Comparison of Ka, KS, and Ka/Ks values across different *GmOPR*s. Ka, KS, and Ka/Ks values for each gene represent the average. (**B**) Comparison of Ka/Ks values for *GmOPR*s across 28 accessions.

To assess the natural selection pressure on *OPR* genes in soybean, the *OPR*s of ZH13 were compared with those of 27 other soybean genomes. This comparison was used to calculate the values of non-synonymous substitutions (Ka), synonymous substitutions (Ks), and Ka/Ks. The range of mean Ka values (0.0001–0.0147) was lower than the range of mean Ks values (0–0.0287). Most Ka and Ks values below 0.02 were lower overall (Figure 3A and Table S5). *GmOPR2* and *GmOPR4* had higher Ka and Ks values, and the expression of these genes had higher mutation rates, possibly due to the experience of lenient natural selection. The average Ka/Ks values ranged from 0 to 1.47, with 86.7% of the Ka/Ks values in the 28 soybean accessions being less than 1, indicating that the overall evolutionary pressure on *GmOPR*s was dominated by purifying selection, but that there were potential positive selection signals as well. The Ka/Ks values of *GmOPR5* in C05, *GmOPR7* in L02, and *GmOPR9* in C02 were higher than 1 (Figure 3B), suggesting that these genes have experienced strong selection only in certain soybean accessions that experienced strong positive selection pressure. In contrast, *GmOPR6* and *GmOPR14* exhibited Ka/Ks values greater than 1 in several soybean accessions, suggesting that these genes may have been subjected to strong positive selection pressure during soybean evolution. It suggests that these genes may be undergoing adaptive evolution, and further experiments are needed to verify whether this is related to biological function.

### 3.4. Cis-Acting Elements Analysis of GmOPRs in the 28 Soybean Accessions

To further study the expression regulation mode of *GmOPR*s, cis-acting elements 2000 kb upstream of *OPR*s in 28 soybean genomes were predicted, analyzed, and compared, and a total of 52 species with 11,972 cis-acting elements were identified, with an average of 428 cis-acting elements in each species (Table S6), which were classified into growth and development. The cis-acting elements were categorized as growth and development, hormone responsive, light responsive, stress responsive, and transcription factor binding.

**Figure 4:**
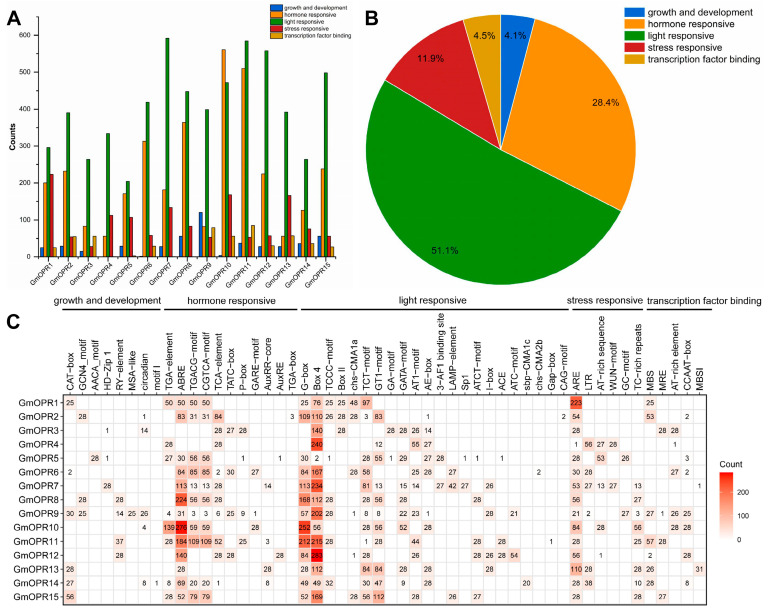
Analysis of cis-acting elements in *GmOPR*s across the 28 soybean accessions. (**A**) Distribution of cis-acting elements among different *GmOPR*s in the 28 soybean accessions. (**B**) Distribution of cis-acting elements within the *GmOPR* gene family across the 28 soybean accessions. (**C**) Number of cis-acting elements in different *GmOPR*s across the 28 soybean accessions.

Light-responsive was the most abundant element in *GmOPR*s, accounting for 51.1% of the total; hormone-responsive was the next most abundant, and growth and development accounted for the least, 4.1% (Figure 4B). The same pattern and some differences in the types and numbers of cis-acting elements existed in different *GmOPR*s (Figure 4A), with *GmOPR10* having the highest number of cis-acting elements, and *GmOPR*s having the highest number of light-responsive elements, except for hormone-responsive elements that were more abundant than light-responsive elements in *GmOPR10*. A relatively large number of growth and development elements were present in *GmOPR9*, and the proportion of stress-responsive genes was larger in *GmOPR1*, *GmOPR4*, and *GmOPR13*, where these genes may exhibit different expression patterns due to the different proportions of cis-acting elements. Anaerobic response elements (AREs) were enriched in *GmOPR1*, abscisic acid response elements (ABREs) were enriched in *GmOPR8* and *GmOPR10*, and MBSI elements associated with flavonoid biosynthetic gene regulation were heavily enriched in *GmOPR13* (Figure 4C). The AACA motif element associated with endosperm-specific negative expression was only present in *GmOPR5*, and the MSA-like element associated with cell cycle regulation was only present in *GmOPR9*. The prevalence of specific cis-acting elements numbering less than 10 in *GmOPR*s suggests that these cis-acting elements are only present in a small proportion of varieties, which may be related to the evolution of varietal adaptation. The cis-acting element types of *GmOPR1*, *GmOPR8*, *GmOPR13*, and *GmOPR15* are conserved, and the absence of specific elements suggests that these genes may be critical for the *GmOPR*s to exercise important functions in gene regulatory networks.

### 3.5. Protein Interaction Network Prediction and Tissue Expression Pattern Analysis

**Figure 5:**
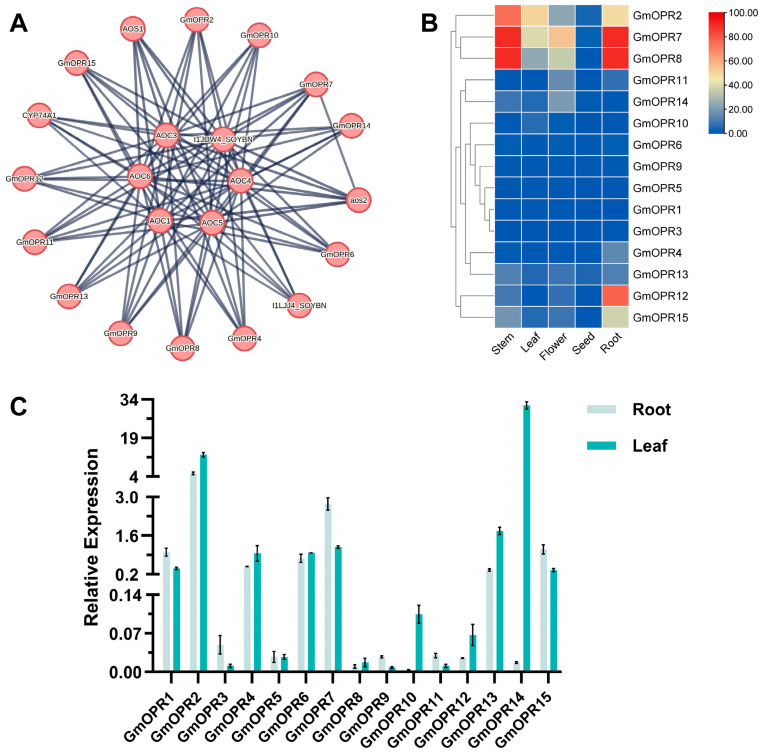
GmOPRs protein interaction network and tissue expression patterns. (**A**) GmOPRs protein interaction network. (**B**) Heatmap of *GmOPR*s expression in different tissues. Standardized FPKM values are shown in the upper right corner. (**C**) Relative expression levels of *GmOPR*s in roots and leaves detected by qRT-PCR experiments. Standard deviations are represented by error bars.

Protein interaction networks can be used to predict functional homologs in sequence homology groups, which is important for studying gene interactions and regulatory relationships. The OPR protein sequence in the ZH13 genome was used as representative input for the STRING database to predict protein interaction networks. I1JDW4_SOYBN had an Allene_ox_cyc domain to judge it as an AOC protein, and CYP74A1 and I1LJJ4_SOYBN have p450 domains; then they may be AOS proteins. The protein interaction network consisted of 22 proteins, including 12 GmOPR proteins, 4 AOS proteins, and 6 AOC proteins (Figure 5A). In the protein interaction network, AOCs are located upstream of GmOPRs, downstream of AOSs, and interact with both GmOPRs and AOSs. Twelve GmOPR proteins participate in protein interaction networks, indicating that these GmOPRs may be crucial members within the *GmOPR* gene family. It is noteworthy that GmOPR7 is not only associated with AOCs but also with AOS2, suggesting that it may play a more important role in the network.

In order to gain a deeper understanding of the expression patterns of different *OPR* genes in soybean tissues. The transcriptome data of different tissues of soybean were retrieved from the SoyOmics database, and the expression profiles of *GmOPR* gene family members in these tissues were subsequently mapped using TBtools software (Figure 5B, Tables S7 and S8). *GmOPR1*, *GmOPR3*, *GmOPR5*, *GmOPR6*, and *GmOPR9* were hardly expressed in various tissues of soybean, and all the *GmOPRs* were expressed at low levels in seeds. *GmOPR7* and *GmOPR8* existed in a similar expression pattern and were highly expressed in the stem and root, followed by the flower and leaf. *GmOPR2* was highly expressed in root, stem, and leaf, while *GmOPR12* and *GmOPR15* were highly expressed in root.

To verify the accuracy of the transcriptome database, qRT-PCR experiments were conducted on roots and leaves for 15 genes within the *GmOPR* gene family (Table S10). Results showed that *GmOPR3*, *GmOPR7*, *GmOPR9*, *GmOPR11*, and *GmOPR15* exhibited significantly higher expression levels in roots than in leaves, while *GmOPR2*, *GmOPR10*, and *GmOPR14* showed significantly higher expression in leaves than in roots (Figure 5C). These findings were consistent with the trends observed in the transcriptome database.

### 3.6. Analysis of GmOPRs Expression Patterns Under Salt Stress

**Figure 6:**
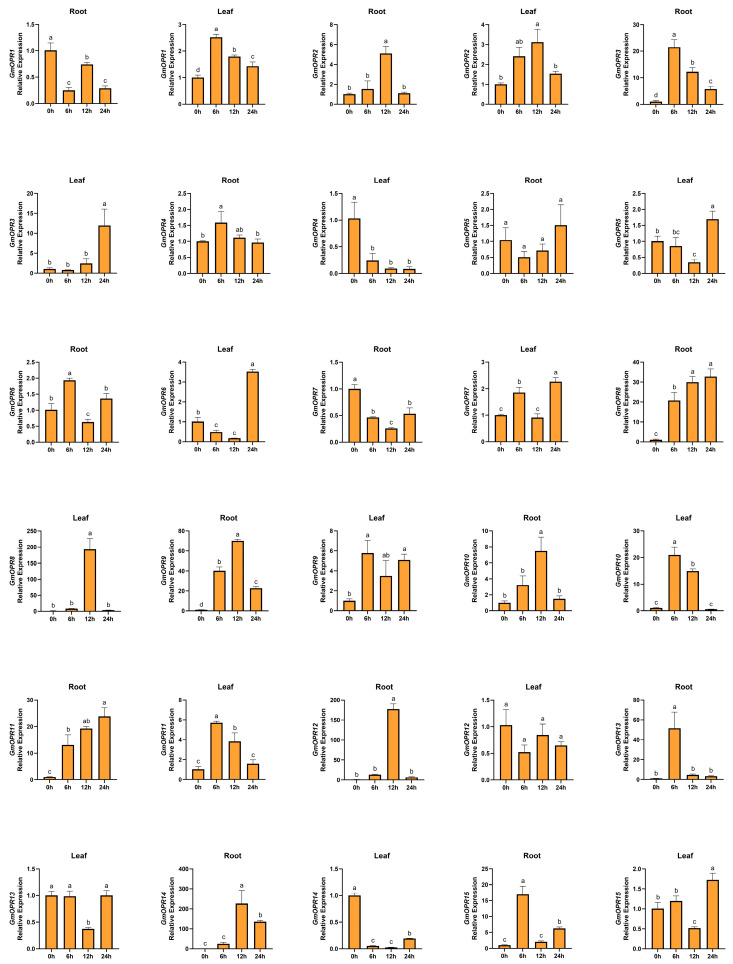
Expression patterns of the *GmOPR* gene family under salt stress. The relative expression levels of 15 *GmOPR*s were detected by qRT-PCR at 0, 6, 12, and 24 h of salt stress treatment. All samples were normalized to the average expression of the *Cons4* reference gene. Standard deviations are indicated by error bars, and different letters denote significant differences.

Using the 0-hour treatment as the control, RNA was extracted from roots and leaves at different time points for qRT-PCR analysis and significance testing. In roots subjected to 6-hour salt stress, *GmOPR3*, *GmOPR6*, *GmOPR13*, and *GmOPR15* showed significant upregulation, while in leaves, *GmOPR1*, *GmOPR10*, and *GmOPR11* exhibited significant upregulation (Figure 6 and Table S10). In roots subjected to 12-hour salt stress, *GmOPR2*, *GmOPR3*, *GmOPR9*, *GmOPR10*, *GmOPR12*, and *GmOPR14* were significantly upregulated, while *GmOPR*2 and *GmOPR8* were significantly upregulated in leaves. In roots subjected to 24-hour salt stress, *GmOPR8* and *GmOPR11* were significantly upregulated, while in leaves, *GmOPR3*, *GmOPR5*, *GmOPR6*, *GmOPR7*, *GmOPR9*, and *GmOPR15* were significantly upregulated. *GmOPR9* expression increased in both roots and leaves across all three time points following salt stress. *GmOPR8* and *GmOPR11* expression in roots increased across all three time points after salt stress. *GmOPR1* and *GmOPR7* in roots, along with *GmOPR4* and *GmOPR14* in leaves, showed decreased expression across all three time points after salt stress. In summary, *GmOPR2*, *GmOPR3*, *GmOPR6*, *GmOPR8*, *GmOPR9*, *GmOPR10*, *GmOPR11*, *GmOPR12*, *GmOPR13*, *GmOPR14*, and *GmOPR15* in roots were upregulated in response to salt stress. In leaves, *GmOPR1*, *GmOPR2*, *GmOPR3*, *GmOPR5*, *GmOPR6*, *GmOPR7*, *GmOPR8*, *GmOPR9*, *GmOPR10*, *GmOPR11*, and *GmOPR15* were up-regulated in response to salt stress. Except for *GmOPR4*, the expression levels of the remaining 14 *GmOPR*s were induced by salt stress in different tissues. This indicates that the *GmOPR* gene family generally responds to salt stress. *GmOPR2*, *GmOPR3*, *GmOPR6*, *GmOPR8*, *GmOPR9*, *GmOPR10*, *GmOPR11*, and *GmOPR15* were up-regulated in both roots and leaves in response to salt stress, indicating their crucial roles in soybean salt stress responses. Notably, *GmOPR1*, *GmOPR7*, and *GmOPR14* exhibited opposite expression patterns in roots and leaves, suggesting tissue-specific functions for these genes. 

## 4. Discussion

Soybean is an important grain and oil crop in the world, and salt stress is one of the main abiotic factors affecting soybean yield, while the area of saline land is increasing year by year, and improving the salt tolerance of soybean can effectively enhance the total soybean yield and soil utilization [57]. Long-term selection of a few high-yielding genotypes during modern breeding has resulted in the loss of a large amount of genetic variation for salt tolerance in cultivated soybeans. Traditional studies based on a single reference genome (e.g., Williams 82) have identified some salt tolerance genes (e.g., *GmSALT3*) [58]. However, there is a systematic omission of germplasm-specific salt tolerance gene members and their allelic diversity, such as important salt tolerance-enhancing genes like *GsWRKY40* and *GsERD15B*, which are not included in the reference genome in wild soybean [59,60], as well as rare protective allelic variants in cultivars. JA is considered an endogenous regulator that plays an important role in plant adversity response, growth, and development, and other life processes [61]. OPR is a crucial enzyme in the jasmonate synthesis pathway. The identification and analysis of *OPR* genes have been extensively studied in many plants, such as *Arabidopsis thaliana* [54,62], rice [63], and maize [64]. However, analysis of the soybean gene family based on multiple varieties has rarely been reported, and research on *OPR* genes in soybean remains relatively scarce.

This study aims to provide a novel approach for soybean gene family analysis by conducting *OPR* family analysis on 28 soybean genomes constructed from three wild soybean accessions, nine local accessions, and sixteen cultivated accessions. It overcomes the limitations of a single reference genome to comprehensively elucidate the composition, genetic diversity, and functional evolution of the *OPR* gene family in soybean, demonstrating the feasibility and superiority of gene family analysis based on soybean multiple genomes. We identified *OPR* genes from 28 soybean genomes and recalibrated their annotations. In total, there were 69 unannotated genes discovered and 36 erroneous genes corrected. By comparing the *OPR* members in multiple genomes and then re-annotating them, the accuracy and reliability of the *OPR* gene members in the multiple genomes were ensured, and a new gene was identified using this method, which was not possible in the previous single-genome gene family analysis. 15 *OPR* genes were identified in the soybean multiple genomes, containing 14 core genes and 1 variable gene, of which four pairs of genes were syntenically related. Only *GmOPR1* was missing in soybean L06, C08, and L04, and the rest of the genes were present in all genomes. The conserved members of the soybean *OPR* gene family indicate that they carry the core biological functions indispensable for species survival. In the analysis of *OPR* gene duplication types, it was shown that WGD/segmental duplication was the main force driving the evolution of the soybean *OPR* family.

*OPR3* is an important gene in the *OPR* gene family, and 12-oxophytodienoate reductase 3 is a crucial rate-limiting enzyme in the plant JA biosynthetic pathway [5], which has been identified and studied in *Arabidopsis thaliana* [54,55] and rice [56]. Soybean has the largest number of *OPR* genes among all species in the phylogenetic tree. By grouping *OPR* genes and analyzing their evolutionary relationships, subgroup VII may be an important subgroup that contains several identified *OPR3* genes, and 4 soybean *OPR3* candidate genes were selected, which can be used for a subsequent in-depth study of their functions. Phylogenetic analysis revealed that the number of *OPR*s increased significantly in soybean. Most *MtOPR*s and *GmOPR*s were found in the same subgroup, and there were significantly more of them than *AtOPR*s. This suggests that *OPR* genes differentiated between the *Leguminosae* and *Cruciferae* families and that large-scale duplications occurred in the *Leguminosae* family.

Selection pressure analyses revealed that the overall evolutionary pressure on *OPR*s was dominated by purifying selection, but there were also signals of potential positive selection. *GmOPR2* and *GmOPR4* may be experiencing lenient natural selection, and *GmOPR5*, *GmOPR7*, and *GmOPR9* are experiencing strong natural selection only in certain soybean varieties that experienced strong positive selection pressure. In contrast, *GmOPR6* and *GmOPR14* exhibited Ka/Ks values greater than 1 in several soybean accessions, suggesting that these genes may have been subjected to strong positive selection pressure during soybean evolution. It suggests that these genes may be undergoing adaptive evolution, and further experiments are needed to verify whether they are related to biological functions. The use of multiple genomes provides a more comprehensive assessment of selection pressures within soybean species, whereas single-genome analysis for interspecies selection pressures does not provide feedback on subtle evolutionary trends in soybean populations. Moreover, the large sample size of multiple genomes can reconcile the significant heterogeneity of selection pressures among individual species, making the results more valid and reliable.

In cis-acting element prediction analysis, traditional analysis relies on the reference genome of a single line, which will miss gene family members and their regulatory sequences that do not exist in that line. The 28 soybean accessions integrate wild type, cultivar, and local species, presenting all genes of the gene family and the 2000 bp region upstream of their promoters, avoiding incomplete analysis of regulatory elements due to the absence of the reference genome. Moreover, by comparing the same gene in the *OPR* gene family of different accessions, it is possible to identify the conserved core cis-acting elements common to all accessions and to discover the variable cis-acting elements that exist only in some accessions. Many variable cis-acting elements, such as chs-CMA2b, TGA-box, etc., are only found in specific varieties and may be linked to varietal trait differences. In contrast, the cis-acting element types in *GmOPR1*, *GmOPR8*, *GmOPR13*, and *GmOPR15* were conserved, and no specific elements appeared, suggesting that these genes may be important and exercise important functions in gene regulatory networks. In addition, *GmOPR10* has a large number of hormone-responsive elements and stress-responsive elements, and its expression was significantly up-regulated in roots and leaves under salt stress, suggesting that *GmOPR10* may enhance the salt tolerance of soybean through various hormone signaling pathways.

Protein interaction network predictions revealed an AOS-AOC-GmOPR regulatory module, which is consistent with existing studies on the jasmonate synthesis pathway [65]. Not only were 12 more important GmOPRs selected, but one particular gene analyzed was GmOPR7, which was also linked to AOS2 compared to other GmOPRs, suggesting that it may play a more unique role in the network and could be functionally investigated by subsequent experiments.

The expression pattern of *GmOPR*s in various tissues showed that some of the *GmOPR*s were hardly expressed in various tissues of soybean, and *GmOPR*s as a whole were hardly expressed in seeds. It is noteworthy that *GmOPR7* and *GmOPR8* showed similar expression patterns, with high expression in stems and roots, followed by flowers and leaves, and these two genes may perform similar functions in soybean and are widely involved in soybean growth and development. *GmOPR12* and *GmOPR15* were specifically and highly expressed in roots, suggesting that these genes are essential for root development and physiological functions. Most *GmOPR*s were validated by qRT-PCR to align with the trends observed in transcriptomic data.

The association between *OPR* gene families and plant salt tolerance has been established in multiple plant species, such as *TaOPR1* in wheat [30], *ZmOPR1* in maize [31], and *AhOPR6* in peanut [32], with mechanisms often linked to the ABA signaling pathway. However, studies on *OPR* genes in salt stress-related research in soybeans remain scarce. This study focused on 15 *OPR* genes identified in the 28 soybean accessions, investigating their expression patterns under salt stress at 0, 6, 12, and 24 h. All 15 *GmOPR*s exhibited altered expression levels in response to salt stress. Except for *GmOPR4*, which showed downregulation in leaves under salt stress but no response in roots, the remaining 14 genes were upregulated in at least one tissue. This indicates the broad involvement of the *GmOPR* gene family in salt stress responses. Notably, *GmOPR1*, *GmOPR7*, and *GmOPR14* exhibited both up- and down-regulation in different tissues, suggesting these genes may perform distinct functions in roots and leaves. Furthermore, *GmOPR3*, *GmOPR8*, *GmOPR9*, *GmOPR10,* and *GmOPR11* showed highly significant up-regulated expression in both roots and leaves under salt stress, indicating a stronger functional association with salt stress responses. Subsequent experiments focusing on the in-depth molecular mechanism analysis of these genes will provide theoretical support for developing salt-tolerant soybean accessions. The situation discussed in this study is a neutral salt represented by NaCl, but real saline environments also contain alkaline salts dominated by Na_2_CO_3_/NaHCO_3_ [66]. And the response mechanisms of plant coping under different salt stresses are also different. In neutral salt stress, the core of the plant response mechanism lies in the activation of the SOS signaling pathway for Na^+^ efflux from the roots and enhancement of salt tolerance in the stem tissues, as well as the enhancement of the reactive oxygen species scavenging system [67]. Alkaline salt stress is resisted by enhancing photosynthetic carbon assimilation and promoting the synthesis of organic acid metabolic modules [68]. Future studies should continue to explore the performance of these genes, whose expression was significantly up-regulated under neutral salt stress, in mixed salinity. This will better assist the actual breeding of soybeans for salinity tolerance.

## 5. Conclusions

This study conducted a systematic bioinformatics analysis of the soybean *OPR* gene family based on the 28 soybean accessions, including gene family members, genomic distribution, duplication events, and synteny, gene structure, conserved motifs, phylogenetic evolutionary relationships, selection pressure, cis-acting regulatory elements, protein–protein interaction networks, and tissue-specific expression patterns. Provides a theoretical basis for a deeper understanding of the structure and function of the *GmOPR* gene family. In addition, further analysis of the expression patterns of the *GmOPR* gene family under salt stress revealed that *GmOPR*s are broadly involved in salt stress responses, with some exhibiting tissue specificity. The relative expression levels of *GmOPR3*, *GmOPR8*, *GmOPR9*, *GmOPR10*, and *GmOPR11* were significantly up-regulated in roots and leaves under salt stress. Furthermore, among the *GmOPR*s, *GmOPR10* has a large number of stress-responsive elements and the most hormone-responsive elements, suggesting that *GmOPR10* may be a key gene that plays a central regulatory role in plant salt stress resistance. This study provides important candidate genes for breeding salt-tolerant soybean varieties. Subsequent experiments can verify its role in salt stress by gene overexpression and CRISPR/Cas9. And observe its role in mixed salt. Thus, the genetic improvement of salinity tolerance in soybean varieties can be realized. Future studies should also focus on the specific mechanism of action so that more genes related to soybean salt stress resistance can be screened and applied to improve soybean salt tolerance. In summary, these findings ensure the accuracy of gene family analysis and reflect the genetic diversity of different soybean accessions, providing a new idea for soybean gene family analysis that should be widely promoted.

## Data Availability

The original contributions presented in this study are included in the article/Supplementary Material. Further inquiries can be directed to the corresponding authors.

## Supplementary Materials

The following supporting information can be downloaded at: https://www.mdpi.com/article/10.3390/biology15010032/s1, Table S1: List of GmOPR genes identified in the 28 soybean accessions and their related information; Table S2: Representative GmOPR gene duplication events; Table S3: The GmOPR motifs in MEME websites; Table S4: Oryza sativa, Arabidopsis thaliana, Medicago truncatula and Glycine max OPR genes in phylogenetic analysis; Table S5: One-to-one orthologous relationships between the OPR gene members in ZH13 and 27 soybean genomes; Table S6: Cis-element analyses of the GmOPR gene promoter regions; Table S7: Expression profiles of GmOPR genes in multiple tissues throughout various developmental stages; Table S8: Expression profiles of representative GmOPR genes in multiple tissues throughout various developmental stages; Table S9: Primers used in this study for qRT-PCR. Table S10: Analyzed qRT-PCR data under salt-treated conditions.
